# Optical Graphene Gas Sensors Based on Microfibers: A Review

**DOI:** 10.3390/s18040941

**Published:** 2018-03-22

**Authors:** Yu Wu, Baicheng Yao, Caibin Yu, Yunjiang Rao

**Affiliations:** Fiber Optics Research Centre, Key Laboratory of Optical Fiber Sensing and Communications (Education Ministry of China), University of Electronic Science and Technology of China, Chengdu 610054, China; yaobaicheng@uestc.edu.cn (B.Y.); yucaibin.0926@163.com (C.Y.)

**Keywords:** microfiber, graphene, highly sensitive, gas sensors

## Abstract

Graphene has become a bridge across optoelectronics, mechanics, and bio-chemical sensing due to its unique photoelectric characteristics. Moreover, benefiting from its two-dimensional nature, this atomically thick film with full flexibility has been widely incorporated with optical waveguides such as fibers, realizing novel photonic devices including polarizers, lasers, and sensors. Among the graphene-based optical devices, sensor is one of the most important branch, especially for gas sensing, as rapid progress has been made in both sensing structures and devices in recent years. This article presents a comprehensive and systematic overview of graphene-based microfiber gas sensors regarding many aspects including sensing principles, properties, fabrication, interrogating and implementations.

## 1. Introduction

Microfibers with sub-wavelength geometry and large index contrast between the fiber core and surroundings [1,2] has become a widely used technique in fiber optics, benefiting from its outstanding properties: evanescent field excitation, electromagnetic confinement, low transmission loss, and coupling convenience [3,4]. Among diverse microfiber applications, optical sensing is very interesting due to its potential of realizing miniaturized fiber optic sensors with small footprint, high sensitivity, fast response, good flexibility, and low power consumption [5,6]. Especially in recent years, people have found that microfibers can work as an ideal substrate for realizing high-performance chemical sensors incorporated with two-dimensional optoelectronic materials, such as graphene or graphene oxide.

Graphene is a unique two-dimensional material composed of carbon in a honeycomb lattice with atomic thickness [7,8], and has spurred remarkable advances ranging from chemical physics and materials science [9,10], to optoelectronics, mechanics, and thermal processes [11,12,13,14]. In photonics, driven by its quasiparticle Dirac fermions obeying a linear dispersion and chiral symmetry [15,16], graphene enables its optical conductivity defined only by the fine structure constant [17], which is with remarkable carrier-density tunability and corresponding surface sensitivity [18,19,20,21]. Consequently, a series of state-of-the-art graphene-based optoelectronic and photonic devices have been investigated, including modulators, fast lasers, detectors, converters, and biochemical sensors [22,23,24,25,26,27,28,29].

Among them, graphene-based gas detection is one of the most creative and successful applications, showing the potential to achieve ultimate sensitivity: single molecule gas detection [30]. Moreover, benefiting from its atomic thickness with ultrahigh conductivity, graphene can also realize remarkable functionalities, such as wearable sensors for smart systems [31,32,33] and switching-sensing devices for high-precision measurements with thermal compensation [34,35].

When deposited on microfibers, graphene interacts with the evanescent field, enabling different kinds of graphene-based microfiber gas sensors. Here we conclude the recent progress in this fiend regarding their sensing principles, fabrication and performances. Typical graphene-based microfiber gas sensors include biconical tapers, gratings, interferometers, coils, ring cavities, and so on. These fiber-optic graphene gas sensors are categorized in both passive (such as interferometric) and active (such as pumping laser-based) techniques, which shows the potential applications in highly-sensitive gas sensing in combining with microfiber and new materials. Although some review articles on graphene-based electrical gas sensors have been published [9,10], there are currently no available review articles on graphene-based microfiber gas sensors. Hence, this article aims to provide an overview of the research and development on graphene-based microfiber gas sensors over the past 5 years. Finally, we summarize with an outlook for challenges and opportunities of the optical graphene gas sensors based on microfibers.

## 2. Principles of Graphene-Based Gas Sensing on Microfibers

**Figure 1:**
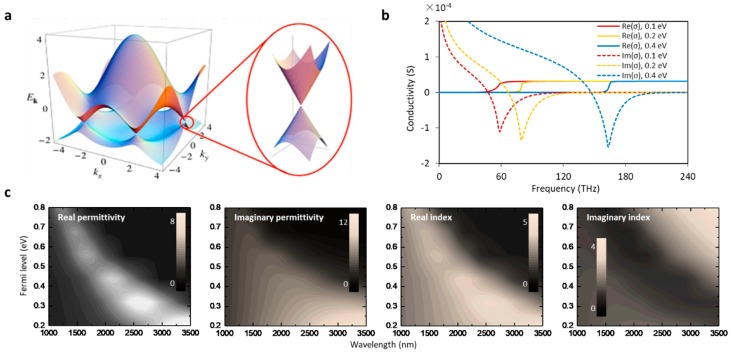
Optoelectronic property of graphene (**a**) Band structure; (**b**) Calculated conductivities varying in Fermi level; (**c**) Calculated permittivity and refractive index, in real and imaginary parts, respectively. Here (a,b) are reproduced from Refs. [36,42], respectively.

The electronic conductive band and valence band crosses at the Dirac point, as shown in Figure 1a [36]. The Fermi level of graphene can be simply described by using the dispersion relation *E_±_*(***κ***) *= ±ħν_F_|**κ**|*, where ***κ*** is the wave vector, *ν_F_* ≈ 10^6^ m/s is the Fermi velocity, and *ħ* is Planck’s constant [37]. When the Fermi level is higher than the Dirac point, graphene is N-doped; otherwise it is P-doped. Accordingly, the optical conductivity of graphene can be written as [38]:

(1) $$\sigma_{g}\left(f,E_{F},\tau ,T\right)=\frac{ie^{2}\left(2\pi f-i/\tau \right)}{\pi \hbar^{2}}\left\{\frac{1}{{\left(2\pi f+\frac{i}{\tau }\right)}^{2}}\int_{0}^{\infty }\varepsilon \left[\frac{\partial f_{d}\left(\epsilon \right)}{\partial \epsilon }-\frac{\partial f_{d}\left(-\epsilon \right)}{\partial \epsilon }\right]d\epsilon -\;\int_{0}^{\infty }\left[\frac{f_{d}\left(-\epsilon \right)-f_{d}\left(\epsilon \right)}{{\left(2\pi f+i/\tau \right)}^{2}-4{\left(\epsilon /\hbar \right)}^{2}}\right]d\epsilon \right\}.$$

Specifically, the intraband conductivity and the interband conductivity can be approximately separated as:

(2) $$\sigma_{g,intra}=\frac{ie^{2}E_{F}}{\pi \hbar \left(2\pi f+\frac{i}{\tau }\right)}$$

(3) $$\sigma_{g,inter}=\frac{ie^{2}E_{F}}{4\pi \hbar }ln\left[\frac{2\left|E_{F}\right|-\hbar \left(2\pi f+\frac{i}{\tau }\right)}{2\left|E_{F}\right|+\hbar \left(2\pi f+\frac{i}{\tau }\right)}\right].$$

The theoretically calculated results are shown in Figure 1b. Here *E_F_* is the quasi Fermi level, directly determined by the external bias. *f* is the optical frequency, *τ* ≈ 10^−13^ s is the carrier relaxation lifetime, *T* is the temperature, *f_d_*(*ϵ*) = {*exp*[(*ϵ-μ*)/*k_B_T*] + 1}^−1^ is the Fermi–Dirac distribution, *ħ* = 1.05 × 10^−34^ J·s, *k_B_* = 1.3806505 × 10^−23^ J/K is Boltzmann’s constant, and *e* = −1.6 × 10^−19^ C is the unit charge. External electric tuning majorly influences the *σ_g,inter_*, which is also directly related to the saturable absorption, which is driven by the photoexcited electron kinetics [39]. Considering the fact that graphene has an atomically thick planar waveguide with such a complex sheet conductivity, we write its effective optical permittivity as

(4) $$\epsilon_{g}=\frac{-Im\left(\sigma_{g,i}\right)+iRe(\sigma_{g})}{2\pi f\Delta },$$

where *Re*(*σ_g_*) and *Im*(*σ_g_*) are the real and imaginary parts of *σ_g_*, respectively. By regarding the graphene monolayer thickness Δ = 0.4 nm, the refractive index of the graphene layer can be derived from [40]:

(5) $${\left\{Re\left(n_{g}\right)+iIm(n_{g})\right\}}^{2}=Re\left(\epsilon_{g}\right)+iIm\left(\epsilon_{g}\right).$$

For media modes with real permittivity, we can calculate the relationship as

(6) $$\{\begin{array}{c} Re\left(n_{g}\right)=\frac{2Re\left(\epsilon_{g}\right){\left(\frac{Re\left(\epsilon_{g}\right)}{2}+\frac{\sqrt{Re{\left(\epsilon_{g}\right)}^{2}-Im{\left(\epsilon_{g}\right)}^{2}}}{2}\right)}^{1/2}-2{\left(\frac{Re\left(\epsilon_{g}\right)}{2}+\frac{\sqrt{Re{\left(\epsilon_{g}\right)}^{2}-Im{\left(\epsilon_{g}\right)}^{2}}}{2}\right)}^{3/2}}{\epsilon_{g,i}}\\ Im\left(n_{g}\right)={\left(\frac{\sqrt{Re{\left(\epsilon_{g}\right)}^{2}+Im{\left(\epsilon_{g}\right)}^{2}}}{2}-\frac{Re\left(\epsilon_{g}\right)}{2}\right)}^{1/2} \end{array}.$$

Figure 1c maps the numerically calculated results of the complex permittivity and complex refractive index. These results directly indicate the optical dispersion of graphene-based microfiber structures, which was experimentally demonstrated by Yao et al. in 2013 [41].

**Figure 2:**
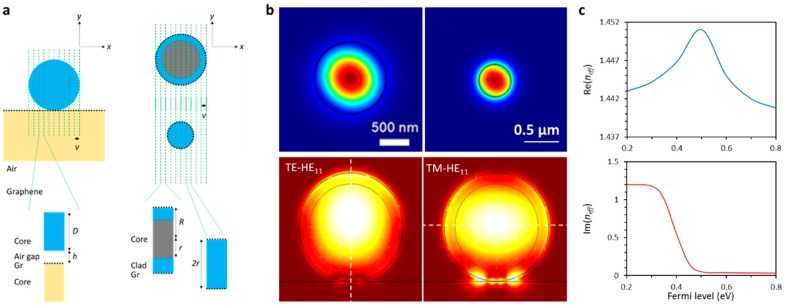
Calculation and simulation of graphene-based microfibers. (**a**) Scheme of the finite element method (FEM) simulation for graphene-based microfibers. (**b**) Simulated electric field intensity distributions. (**c**) Calculated effective index of a graphene-based microfiber, relying on the Fermi level of graphene. Here simulations in (**b**) are reproduced from Refs. [43,46].

Once incorporated with a microfiber, the graphene performs as a part of the cladding, and can influence the optical transmission via mode effective refractive index modulation. According to different graphene–microfiber hybrid geometries, the mode field distributions and complex effective index *n_eff_* can be numerically simulated or calculated via finite element method (FEM) [43,44]. Figure 2a illustrates the idea of modelling the graphene-based microfibers for effective index approximation. Figure 2b provides examples of the spatial distributions of electric field intensity (fundamental mode) for different types of graphene-based microfiber structures. Upper panels: graphene-wrapped microfiber with core diameter ≈ 0.5 µm and 1 µm [43,45], lower panels: graphene-wrapped microfiber with core diameter ≈ 8 µm while cladding thickness ≈ 2 µm [46]. The mode effective index of a hybrid waveguide is also influenced by the optoelectronic dynamics of graphene. Figure 2c plots the simulated “*n_eff_* vs. |*E_F_*|” correlation of a graphene-based microfiber with core diameter ≈ 8 µm. It can be seen that by changing the Fermi level of graphene, both the transmission phase and the transmission loss of a graphene-based microfiber could be modified remarkably. Such a mechanism can not only be applied to sensing, but has also been widely used for fast optical modulation [47,48].

It is known that the electromagnetic field distributed along microfibers obeys the Bessel equations in cylindrical coordinates [49], which has a general solution

(7) $$E=Ae^{\frac{\omega Im(n_{eff})z}{c}}e^{i\left[\frac{\omega Re(n_{eff})z}{c}+t\right]},$$

where *A* is the optical amplitude, *Re*(*n_eff_*) and *Im*(*n_eff_*) are the real and imaginary parts of the graphene-based microfiber, *z* is the transmission distance, *t* is the time delay, c is the light velocity in vacuum, and *ω* is the optical frequency. As a conclusion, the Fermi-level modification of graphene would modulate both the output phase and output power of the microfiber. In optical nonlinear processes, such an effect also alters the phase-matching and nonlinear threshold [50].

**Figure 3:**
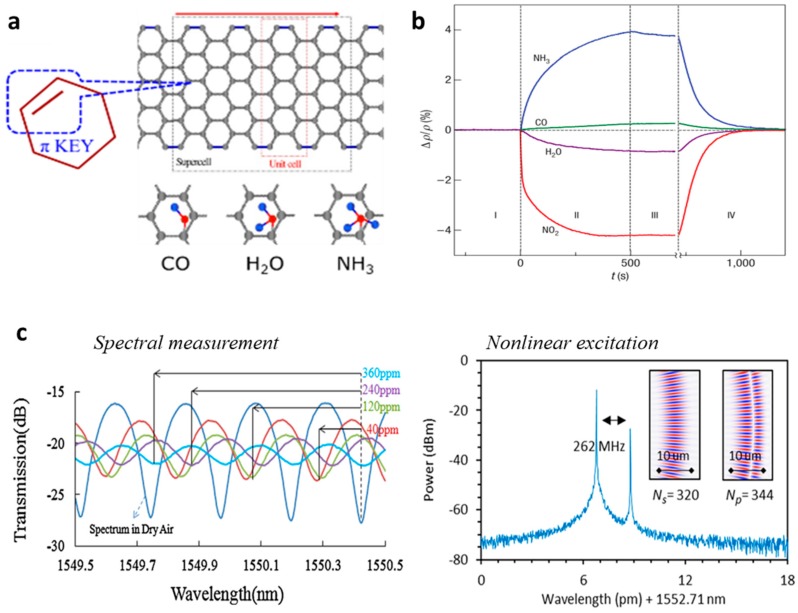
Mechanism of graphene-based electric and optical gas sensors. (**a**) Gas molecules adsorbed on graphene. (**b**) Detecting gas molecules adsorbed on graphene electrically. (**c**) Method to detect gas molecules adsorbed on graphene optically. Here (**a**–**d**) are reproduced from Refs. [30,51,52,53], respectively.

When gas molecules are adsorbed on the surface of graphene, the Fermi level of graphene is tuned [54,55]. Figure 3a shows chemical connections between graphene and gas molecules schematically. For pristine graphene film, the absorption of polar molecules is dominant, hence pristine graphene had been demonstrated with high electronic sensitivity to polar gases such as NH_3_, NO_2_, and H_2_O. It is worth pointing out that for graphene oxide (GO) or other functionalized graphene, non-polar gas such as H_2_ is also detectable [56]. Figure 3b plots the measured “gas adsorption vs. conductivity” for graphene film, reported in Ref. [30]. As expounded above, the “gas adsorption” and the “conductivity-index relationship” of graphene form the basis of the optical graphene gas sensing. By using microfibers, the gas adsorption can be detected via optical interference, power metering, or nonlinearity excitation, as shown in Figure 3c.

## 3. Design and Fabrication of Graphene-Based Microfiber Structures

Since the first graphene-based microfiber optical gas sensor reported in 2012 [57], a variety of graphene-microfiber hybrid structures have been investigated for gas sensing applications. Their fabrication process can be summarized with the following three steps: (1) microfiber fabrication; (2) graphene growth; (3) graphene-microfiber installation.

**Figure 4:**
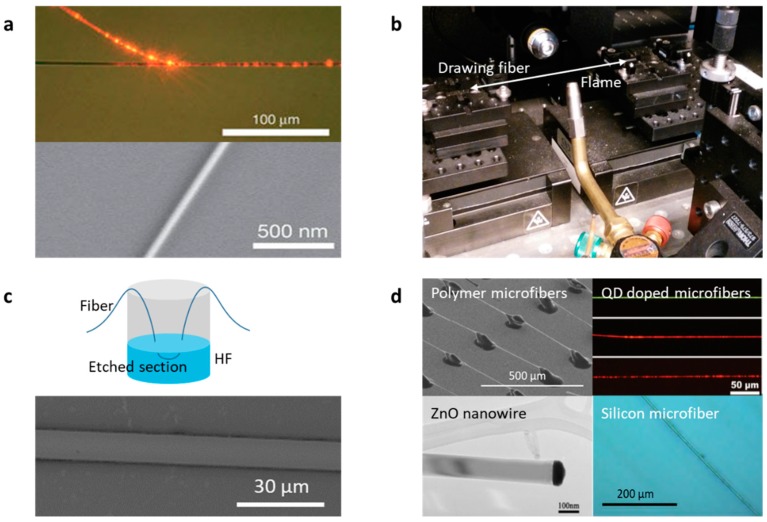
Fabrication of microfibers. (**a**) Pictures of silica microfibers. (**b**,**c**) Setups for fusing & drawing glass microfibers. (**d**) Microfibers besides glass materials. Here pictures of (**a**,**d**) are reproduced from Ref. [1] and Refs. [62,63,64,65,66], respectively.

Typically, microfibers are fabricated from commercial glass fibers or bulks, by using the fusing & drawing method [1], which can be controlled either manually or automatically. Figure 4a shows the microscopic pictures of typical microfibers. The heater source can be either a flame (usually a hydrogen flame), an electrical heater, or a laser-heated tube [58,59,60]. Figure 4b demonstrates a typical setup for fusing & drawing microfibers from glass fibers. Optical loss of a microfiber is mainly determined by its taper quality. Very recently, taking advantage of the “flame-brushing” technique, high-quality silica micro-nano fibers with diameters in the range of 800 nm to 1.3 µm, unevenness < 5 nm, and waist length larger than 30 cm have been achieved [61].

Sometimes one not only wants a microfiber-guiding evanescent field, but also hopes to keep the inner microstructures of the original fiber (such as photonic crystals and Bragg gratings); then, chemical etching becomes a choice [67,68]. For example, for silica-based fiber etching, hydrofluoric acid is commonly used as the corrosive agent. Compared to microfibers fabricated by fusing & drawing, the chemically etched microfibers have larger average diameters, usually in the range of 8–12 µm. Figure 4c shows this method and pictures of the chemically etched microfiber samples. In addition, for further specific purposes, microfibers are not only fabricated from glass materials, but also other materials such as polymers, silicon, and metal-oxides, by using diverse means such as thermal process, chemical growth, and self-assembly [62,63,64,65,66]. Figure 4d shows examples of these special microfibers.

**Figure 5:**
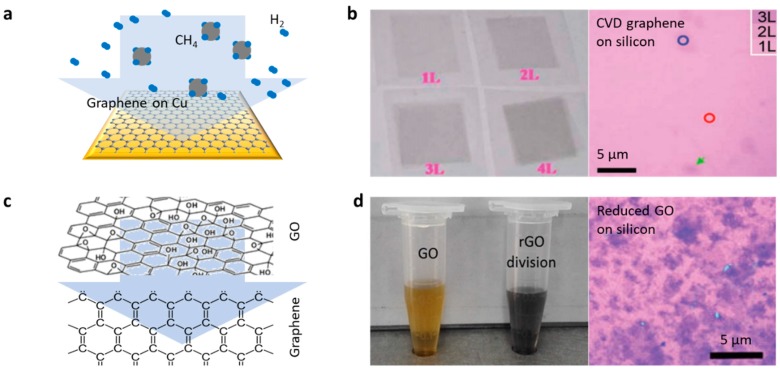
Graphene fabrication. (**a**) Graphene grown by using chemical vapor deposition (CVD) method. (**b**) Pictures of graphene samples fabricated by using CVD method. (**c**) Graphene reduced from graphene oxide (GO) in liquid. (**d**) Picture of GO and reduced GO (rGO) dispersions, and the deposited reduced GO film on silicon substrate. Pictures in (**b**) are reproduced from Refs. [71,72].

The chemical vapor deposition (CVD) and wet transfer techniques provide inch-level large-scale monolayer graphene for incorporation with microfibers [69,70,71,72]. Figure 5a illustrates a schematic view of a growing graphene film on a copper foil. Graphene on Cu is grown by the decomposition of CH_4_ gas in a dilute H_2_ environment over the surface at 1000 °C. With the exposure of Cu foil in a CH_4_/H_2_ environment, the nucleation of graphene islands starts taking place randomly, eventually aggregating into a continuous graphene film. Figure 5b shows the optical image and pictures of CVD graphene film samples. CVD graphene has good uniformity in inch-size large-scale, which has been widely used in optoelectronic devices, via the wet transfer technique. Graphene films can also be deposited on fiber structures directly by reducing from GO in liquid. Figure 5c shows the chemical structures of GO and graphene; there are many functional groups containing oxygen connecting the carbon atoms [73]. Figure 5d shows pictures of liquid dispersions of GO and graphene reduced from GO [74]. Compared to CVD graphene films, the reduced GO film usually has more defects, but the deposition of the GO film does not need an additional transferring process, which is more convenient for implementation. In recent years, by optimizing the solution-based reduction method, large-area GO films with acceptable uniformity can be obtained [75].

**Figure 6:**
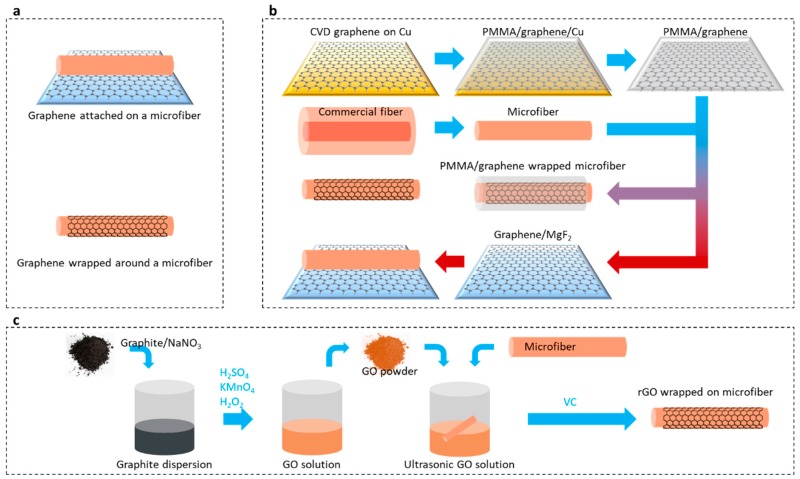
Fabrications. (**a**) Two types graphene-based microfiber structure. (**b**) Fabrication flow of both the graphene-attached microfibers and the graphene-wrapped microfibers, based on CVD graphene. (**c**) Fabrication flow based on GO reduction.

Graphene can be either attached on or wrapped around a microfiber, as schematically shown in Figure 6a. Figure 6b sketches the fabrication flows of a graphene-based fiber structure by using CVD technique [41]. For graphene attached on microfibers, graphene is transferred to a low refractive index substrate; afterwards, microfibers are put on the graphene, kept contacted. Such a van der Waals contact is firm and stable [41]. For graphene-wrapped microfibers, a substrate to carry graphene is unnecessary. The wet transfer of a CVD graphene film is done as follows: spin-coating a layer of polymethyl methacrylate (PMMA) on the surface of the graphene, forming the PMMA/graphene/Cu sandwich-like hybrid; dissolving the Cu under graphene by using FeCl_3_ solution; covering the PMMA/graphene flexible film on a substrate or wrapping it on a microfiber; removing the PMMA by using acetone, leaving only graphene remaining.

Figure 6c shows the process of depositing a reduced GO film on the surface of a microfiber [74]. The process involves oxidizing graphite powder to GO by using strong oxidants such as potassium permanganate; immersing a microfiber in the GO dispersion liquid; reducing the GO to be graphene by using a reductant such as vitamin C; and optimizing the film formation by controlling the temperature and the reduction time.

**Figure 7:**
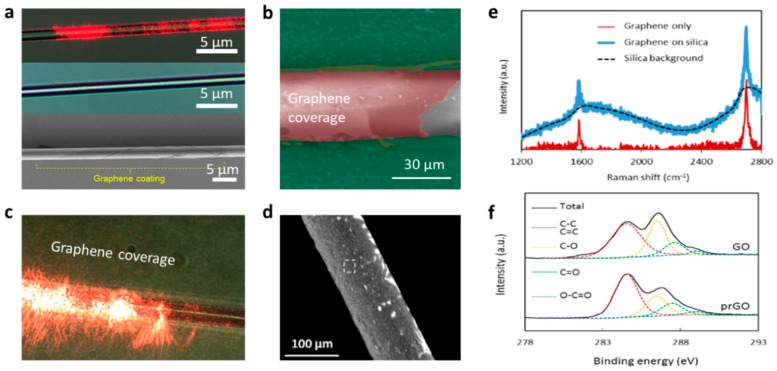
Characterizations of graphene-based microfiber structures. (**a**) Visible scattering, optical microscope, and SEM pictures of a graphene-wrapped hot drawn microfiber. (**b**) A false-colored SEM picture of a graphene-wrapped microfiber etched chemically. (**c**) Light scattering enhancement due to the graphene. (**d**) SEM of an etched microfiber covered by reduced GO. (**e**) Raman spectra. (**f**) Measured X-ray photoelectron spectroscopy (XPS) results. Here figures (**a**–**d**) are reproduced from Refs. [45,46,74,76], respectively.

To characterize the quality of graphene on the microfibers, Raman spectroscopy [77,78,79], scanning electron microscopy (SEM), scattering measurement, and X-ray photoelectron spectroscopy (XPS) [80] are commonly applied. Figure 7a–d show the pictures of graphene-based microfibers, measured by optical microscopy and SEM. One can check the quality of the microfiber-graphene incorporation based on these images. Figure 7e plots a typical Raman spectrum of graphene on a fiber structure. Commonly, high-quality graphene on microfibers should have an ignorable D peak, and a G/2D ratio 0.3~0.5; the locations of the G peak and the 2D peak are influenced by the graphene doping. Commonly, CVD graphene is pure in chemistry, while graphene film reduced from GO may contain more functional groups, such as –OH and –COOH. These functional groups can be extremely useful for specific sensing applications; XPS is helpful to check them. Figure 7f plots the XPS of CVD graphene and reduced GO. For better deposition on microfibers, the reductions are usually controlled to make a C:O ratio > 4 [74].

## 4. Graphene Gas Sensors with Microfibers

In recent years, graphene gas sensors with microfibers develop rapidly, some of them are also reviewed in Refs. [81,82,83,84]. The roadmap demonstrates that the sensitivity of these graphene-based microfiber gas sensors increases from parts per kilo (ppk) to part per billion (ppb) by gradually optimizing the sensing structure.

**Figure 8:**
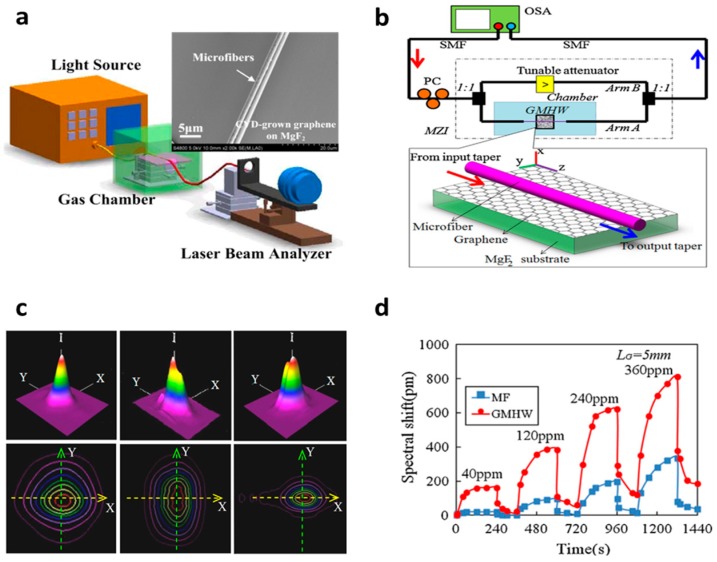
Optical gas sensors based on graphene attached on microfibers by using (**a**) mode-field analysis and power metering; (**b**) MZI interferometer; (**c**) Sensing performance of the graphene-based acetone gas sensor; (**d**) Sensing performance of the graphene-based MZI sensor for NH_3_ gas detection. Here the results are reproduced from Ref. [52,84].

Based on the graphene-attached microfiber scheme, we reported gas sensors based on both optical intensity detection and interferometric demodulation by using mode field analysis or Mach-Zehnder Interferometer (MZI) [52,84]. Figure 8a,b show their implementations. A microfiber with a length of several centimeters and ≈1 µm diameter was used to couple light interacting with graphene. Such a scheme is similar to a graphene-based D-shaped fiber [85,86], but is easier to manipulate. In Ref. [84], we illustrated that the polarization-dependent transmission of the graphene-attached microfiber was sensitive to gas adsorptions, especially large molecules. As Figure 8c shows, the adsorption of acetone gas molecules would dramatically dampen the optical transmission; for example, for acetone gas with a concentration of 1150 ppm, the transmission loss increased over 3 dB, the maximum sensitivity of this structure was about 0.3 dB/ppk.

The regeneration time for the acetone gas detection was in the rage of several minutes. The interferometer scheme was a big step forward to achieve a much higher sensitivity. Figure 8d shows the performance of the graphene-microfiber-based MZI for NH_3_ gas detection [52]. It illustrated both high sensitivity and fast response. For NH_3_ trace detection, 0.3 ppm resolution and 0.5 s response delay was achieved.

**Figure 9:**
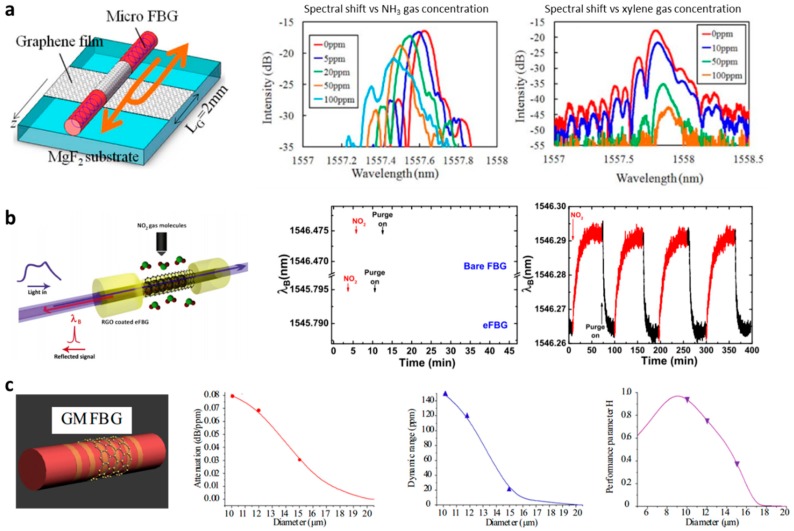
Graphene-based micro fiber Bragg grating (GMFBG) gas sensors (**a**) for NH_3_ gas detection, (**b**) for NO_2_ gas detection. (**c**) Diameter optimization of the micro FBG gas sensors. Figures in (a–c) are reproduced from Refs. [87,88,89].

Similarly, Figure 9b shows the experimental results [87]. Sridevi et al. chose reduced GO rather than CVD graphene, getting better selectivity for NO_2_ gas. A sensitivity of 0.5 ppm was achieved in this work. As the reduced GO film was thicker and fluffier than the CVD graphene, its response time was limited to minute-level. In this type of sensor, it is also important to enhance the mode distribution ratio out of the core and optimize the microfiber diameter (or the thickness of the remained cladding), as discussed by Zhang et al. in Ref. [88]. Figure 9c replots the experimental results: a larger microfiber diameter brought lower sensitivity, but also lower attenuation and larger dynamic range. Considering a composite index *H* ~ *ln*(*S*)l*n*(*D*)/*A*, the best diameter could be around 10 µm for graphene-wrapped silica fiber-Bragg-gratings (FBGs) with standard 8 µm core.

Since the scheme of graphene-microfiber attachment spatially limits the light–graphene interaction, since 2014, graphene-wrapped microfiber structures have become a trend. For instance, based on graphene-wrapped micro fiber Bragg gratings (MFBGs), the footprint of the sensors can be dramatically miniaturized. Wu et al. [89] and Sridevi et al. [87] reported ultrasensitive sensors based on MFBGs for NH_3_ gas sensing and NO_2_ gas sensing, respectively. As shown in Figure 9a, Wu et al. covered a monolayer of graphene around a chemically etched MFBG. The reflection peak of the MFBG was determined by the equation *λ_p_* = 2*n_eff_Λ*, where *Λ* is the MFBG period. The gas adsorption based on *n_eff_* modulation was measured by detecting the spectral shift of the MFBG. The maximum sensitivity of the CVD graphene-coated MFBG reached 0.2 ppm for NH_3_ gas and 0.5 ppm for xylene gas, respectively.

**Figure 10:**
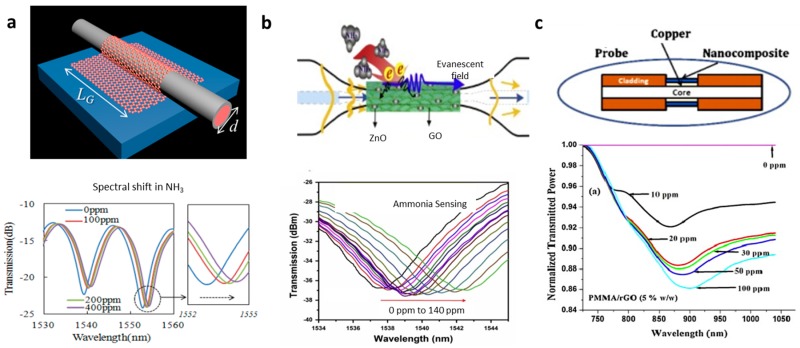
Optical gas sensors based on graphene-wrapped microfiber structures. (**a**) NH_3_/H_2_O sensor based on CVD graphene-microfiber multimode interferometer, (**b**) NH_3_ gas sensor based on ZnO-GO-covered graphene-microfiber multimode interferometer, (**c**) NH_3_ gas sensor based on SPR on a PMMA/graphene/Cu-covered microfiber. Here the figures are reproduced from Refs. [46,91,92].

Another method to enhance light–graphene interaction in graphene-based microfiber structures is to excite high-order mode propagation with larger mode-field area or to excite plasmons. In 2014, Yao et al. demonstrated a graphene-based microfiber multimode interferometer, as shown in Figure 10a [46]. In this structure, the HE_21_ mode is more sensitive to local refractive index alteration than the in-core HE_11_ [90], hence its interference Free Spectrum Range (FSR) could be tuned by gas adsorption, resulting in a spectral resonance dip shift. In a sensing experiment, ~0.1 ppm for NH_3_ gas detection and ~0.2 ppm for H_2_O vapor detection were achieved. By using GO-ZnO film, Hu et al. also recently realized a NH_3_ gas sensor, demonstrating sub-ppm sensitivity, as shown in Figure 10b [91]. This GO-ZnO incorporated interferometric optical microfiber illustrated very high selectivity to NH_3_ gas. Additionally, in 2014, Mishra et al. also reported a graphene Surface Plasma Resonance (SPR) sensor based on microfibers for sensing NH_3_ gas [92]. Mishra et al. applied PMMA/graphene/Cu hybrid membrane directly to enhance the plasmons on Cu and the gas adsorption on the surface of the PMMA/graphene. It demonstrated both good sensitivity (ppm level) and relatively large dynamic range (hundreds of ppm).

**Figure 11:**
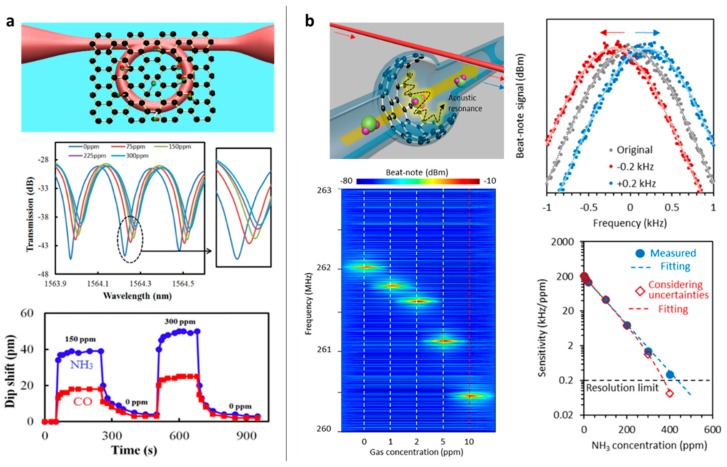
Design and performance of microfiber integrated graphene gas sensors based on resonators. (**a**) NH_3_ gas sensor based on GO deposited microfiber knot, (**b**) NH_3_ gas sensor based on graphene-enhanced Brillouin optomechanics. Figures are reproduced from Ref. [53,93].

Limited by the spectral resolution of the optical interference and the linear loss of the passive devices, sub-ppm seemed to be the limitation for a graphene-based microfiber gas sensor. Taking advantage of high *Q* resonance, the interferometric resolution can be effectively improved. Yu et al. made such an attempt by building a GO-deposited microfiber knot resonator [93]. Figure 11a shows the structural diagram. In this study, the GO film covered the whole microfiber resonator, limiting the *Q* factor. In future investigations, by optimizing the graphene coverage region [94], the resolution and detection limit can potentially be further improved.

Moreover, in 2017, based on graphene-enhanced Brillouin scattering, a microfiber interrogated Whispering Gallery Mode ptomechanical gas sensor was realized. Figure 11b shows the design and the sensing performance. It reported an unprecedented high sensitivity (1 ppb) for NH_3_ gas detection, which is a globally leading number comparable to other advanced techniques [95,96]. Such a remarkable breakthrough revealed the “electron–phonon–photon” interaction in the graphene-based optomechanical resonator, going beyond all the conventional graphene-based optical or solid-state sensors. Optomechanical resonance was generated via Brillouin phase matching and nonlinear gain:

(8) $$\frac{f_{M}}{v_{A}}=\frac{2\pi f_{p}n_{p}}{c}-\frac{2\pi f_{c}n_{c}}{c},$$

(9) $$g_{B}\propto \frac{4\pi^{2}\gamma_{e}{}^{2}n_{p}}{\gamma_{e}c\lambda_{p}{}^{2}\rho_{0}v_{A}\Gamma_{B}},$$

where *v_A_* is the acoustic velocity, *c* is the light velocity in vacuum, *n_p_* and *n_s_* are the effective indexes of the pump mode and the generated Stokes mode, *f_p_* and *f_s_* are the pump frequency and the Stokes frequency, *γ_e_* is the electro-strictive coefficient, and *Γ_B_* is the lifespan of the phonons. Gas adsorption on the reduced GO film enables an RF spectral shift 200 kHz/ppm, while the uncertainty of the Brillouin optomechanical generation is only 200 Hz, due to the extremely high *Q* factor (10^6^). Moreover, such an optomechanical micro-resonator kept an exceptional dynamic range from 1 ppb to 400 ppm, crossing over five orders.

The above review summarizes the progress in optical graphene gas sensors based on microfibers. By optimizing the optical sensing structures, this type of sensor has made significant advancements in sensitivity, which is summarized in Table 1. Benefitting from the advancements in novel optical detection methods, the current performance of microfiber-based optical graphene gas sensors has been comparable to the state-of-the-art gas detection techniques, as displayed in Table 2.

## 5. Conclusions and Outlook

In this article, we review the principles, fabrications, implementations, and performances of optical graphene gas sensors with microfibers, which have attracted intense interest in research and development and play an important role in industry. The mode of “graphene and microfiber” has become a widely used platform for—but not limited to—gas detections. In this way, higher sensitivity and better selectivity are constant pursuits, while still remaining challenges. On one hand, to increase the sensitivity, more and more new optical mechanisms and techniques are being reported, such as graphene-based laser sensing [98], high-order nonlinearity-based enhancement [42], and plasmonic sensing in the Mid-Infrared Range to THz region [20]. On the other hand, determined by the graphene’s nature that it can interact with any gas molecule, most of the above optical gas sensors are focused on polar gas sensing, such as NH_3_, H_2_O, or NO_2_, and these sensors lack selectivity. Towards the realization of graphene-based microfiber sensors for other gas detection, the graphene material itself would be further functionalized, such as by using element-doped films [99], graphene grains [100], or fluorescent resonance energy transformation technology [74]. With the progress in both the microfibers and graphene materials, there is no doubt that more graphene-based fiber-optic gas sensing structures and devices can be foreseen to meet the practical application requirements.

## Figures and Tables

**Table 1:** Major progress in the optical graphene gas sensors based on microfibers.

Year	Sensor Structure	Target Gas	Performance	Reference
2012	Microfiber attached on graphene	Acetone	Sub-ppk sensitivity	[57]
2014	Graphene/gold coated on microfiber for SPR	NH_3_	1 ppm sensitivity	[92]
2014	Graphene-coated microfiber interferometers	NH_3_, H_2_O	Sub-ppm sensitivity Fast response	[46,89]
2016	Reduced GO coated on microfiber Bragg gratings	NO_2_	500 ppb sensitivity~100% recoverability	[87]
2017	Reduced GO-based optomechanic microresonator	NH_3_	1 ppb sensitivity five orders dynamic range	[53]
2018	ZnO-functionalized GO coated on a microfiber multimode interferometer	NH_3_	Sub-ppm sensitivity High selectivity	[91]

**Table 2:** Major progress in optical graphene gas sensors based on microfibers.

Sensor Type	Max Sensitivity	Dynamic Range	Response Time	Reference
Photothermal spectroscopy	2 ppb	six orders	minutes	[96]
Graphene-based SPR on fiber	1 ppm	NG	minutes	[92]
Ultrasensitive plasmonic sensors based on metal	~ppm	NG	NG	[97]
Visible spectroscopy	5 ppb	NG	minutes	[95]
Microfiber-based graphene optomechanic resonator	1 ppb	five orders	seconds	[53]
